# Detection of *N*^3^-methylcytidine in RNA at single base resolution using Protoscript II to read through and generate high mutation rates

**DOI:** 10.1002/jccs.70259

**Published:** 2026-08-04

**Authors:** Qing Dai, Kinga Pajdzik, Caraline Sepich-Poore, Xiaoyang Dou, Hui-Lung Sun, Chang Ye, Li-Sheng Zhang, Boyang Gao, Chuan He

**Affiliations:** 1 Department of Chemistry, The University of Chicago, Chicago, Illinois 60637, USA; 2 University of Chicago Medical Scientist Training Program, The University of Chicago, Chicago, Illinois 60637, USA; 3 Department of Biochemistry and Molecular Biology, The University of Chicago, Chicago, Illinois 60637, USA; 4 Division of Life Science, The Hong Kong University of Science and Technology (HKUST), Kowloon, Hong Kong SAR, China; 5 Howard Hughes Medical Institute, The University of Chicago, Chicago, Illinois 60637, USA

## Abstract

*N*^3^-methylcytidine (m^3^C), an RNA modification involving methylation of cytidine at the Watson-Crick-Franklin edge, has been identified in human tRNA, mitochondrial tRNA (mt-tRNA), and mRNA. In tRNA and mt-tRNA, it has been shown to play important roles in tRNA structure and translation. Here we describe a new approach which utilizes the high readthrough and mutation rates of Protoscript II (PSII) at m^3^C sites in combination with AlkB demethylation to detect m^3^C at single-base resolution. We first validate the method on synthetic probes. Then, using this method, we successfully identify known m^3^C sites on tRNA and mt-tRNA, as well as one new candidate tRNA m^3^C site. We further show that none of the m^3^C sites identified in cytosolic tRNA are likely METTL8 substrates. In contrast, the two m^3^C sites detected in mt-tRNA are sensitive to *METTL8* knockdown (KD) and over expression (OE), in line with their previous characterization as METTL8 substrates. These results demonstrate that our method can be used to investigate changes in the m^3^C level in many sites using an unbiased approach. Overall, this study introduces a convenient high-resolution method for m^3^C mapping with high readthrough rates, paving the way for future mechanistic studies on the biological function of m^3^C and its effector proteins.

## INTRODUCTION

*N*^3^-methylcytidine (m^3^C) is an RNA modification involving methylation of cytidine at the Watson-Crick-Franklin face (Figure 1A). Its chemical properties allow it to confer important functional consequences. Specifically, the location of the methylation on m^3^C causes significant disruption of the Watson-Crick-Franklin C:G pair stability, and hinders base pairing discrimination between C:G and mismatched base pairs.^1^ Increased base pair mismatching can cause destabilization of secondary structure, as well as stopping or mutations during reverse transcription.^1^

m^3^C has been found in human tRNA,^2^ mitochondrial tRNA (mt-tRNA),^2–5^ and mRNA.^2^ It has been most extensively studied in tRNA and mt-tRNA, and remains understudied in RNA types with lower abundance, in part due to much lower m^3^C modification levels documented outside of tRNAs (e.g., 2–4 × 10^−5^ m^3^C/C in human mRNA).^2^ It is present at position 32 of several cytoplasmic and mitochondrial tRNA isoacceptors, where it has been shown to affect tRNA structure and translation.^6^ m^3^C sites have also been identified at other sites in serine, leucine, and methionine isoacceptors.^6^

Several m^3^C writer and eraser proteins have been characterized, and m^3^C can also be introduced by direct, non-enzymatic methylation by endogenous and environmental agents.^7^ In humans, ALKBH1 and ALKBH3 have been shown to demethylate m^3^C-modified RNAs, including mRNA and tRNA.^6,8,9^ There exist four known m^3^C methyltransferase-like proteins in humans: METTL2A, METTL2B, METTL6, and METTL8. In human tRNA, METTL2A and METTL2B install m^3^C_32_ in threonine isoacceptors (*Hs*tRNA^Thr(AGU/CGU/UGU^), and METTL6 introduces m^3^C_32_ in serine isoacceptors (*Hs*tRNA^Ser(AGA/CGA/UGA/GCU)^).^2,10,11^ The writer(s) for m^3^C in leucine and methionine tRNAs, and for some positions in serine tRNAs, remain unknown.^6^ METTL8 was originally presented as a possible mRNA m^3^C methyltransferase, with evidence showing m^3^C levels on mRNA to be significantly lower in liver samples from *METTL8* knockout mice compared to wild-type controls.^2^ However, recent studies have characterized it as the writer for m^3^C_32_ in mt-tRNA^Thr^ and mt-tRNA^Ser(TGA)^.^3–5^ These studies have shown evidence of important METTL8 functions in processes such as mt-tRNA folding,^4^ mitochondrial translation,^3^ and respiratory chain activity.^5^ They have also shown that the most highly expressed isoforms in human cells contain a mitochondrial targeting signal (MTS) and localize to mitochondria^5^. However, isoforms lacking the MTS are also detectable^5^; moreover, when the MTS is removed, METTL8 accumulates in the nucleus.^4,12^ The roles and substrates of *METTL8* isoforms lacking the MTS remain under-characterized.

To investigate m^3^C sites and the functional impact of m^3^C effector proteins on both tRNA and less abundant RNAs, it is critical to map m^3^C locations accurately. Mass spectrometry techniques provide quantitative information about m^3^C levels in bulk RNA, but they do not provide information about specific location(s).^6^ Primer extension assays can investigate modification(s) on specific target sites, but they cannot perform unbiased site discovery.^6^ Although m^3^C antibodies are commercially available, making it possible to profile m^3^C in immunoprecipitation-based sequencing methods,^15^ such methods cannot provide single base resolution. Several next-generation sequencing-based methods have been developed, such as DM-tRNA-seq,^16^ DAMM-seq,^17^ ARM-seq,^18^ mim-tRNA-seq,^19^ AlkAniline-seq,^20^ and HAC-seq.^15^ Some of these methods involve steps that cause extensive RNA degradation, while others identify sites by truncation rather than readthrough with mutation, or use enzymes known to have high background mutation rates, and none of these methods have been used to report m^3^C sites on mRNA, even though mRNA m^3^C has been detected by mass spectrometry.^2^

We endeavored to develop a convenient method for mapping m^3^C which could be used to study known tRNA and mt-tRNA m^3^C sites, and which could potentially be applied to detect sites with lower modification fractions or sites on RNA types with lower abundance without causing significant RNA degradation. We reasoned that since m^3^C affects base pairing, we could identify an enzyme that could read through the modification with high mutation rates at m^3^C and low mutation rates at other C modifications, allowing specific detection of m^3^C sites at single base resolution, and we found Protoscript II to be a promising candidate. Here, we describe the development of a new m^3^C sequencing method utilizing these capabilities of Protoscript II paired with AlkB demethylation and its application in experiments detecting the known m^3^C sites in tRNA and mt-RNA, as well as a pilot experiment using mRNA.

## RESULTS

We first confirmed the presence and abundance of m^3^C in multiple HeLa cell RNA types. This comprised purifying mRNA, chromatin-associated RNA (caRNA), and small RNA (smRNA) from HeLa cells, followed by LC-MS/MS, which revealed that the m^3^C levels in mRNA were about three orders of magnitude lower than in small RNA, which includes tRNA (Figure 1B). Although the mRNA m^3^C level was low, it was similar to that previously reported in HCT116 cell mRNA.^2^ The m^3^C level in caRNA was undetectable in our experiment (Figure 1B). Levels of m^5^C (Figure 1C) and m^6^A (Figure 1D) were measured for each RNA type in the same experiment as references and were found to be in accordance with previously published values [CITE].

Given mass spectrometry provided evidence that m^3^C consistently exists in the aforementioned RNA types, we endeavored to screen reverse-transcriptases and compare their ability to read through and generate mutations at m^3^C sites to determine their relative usefulness in mapping m^3^C. Previously, wild type (WT) HIV reverse transcriptase (HIV RT) has been used for m^3^C detection since it can partially read through m^3^C sites and generate mutations.^21^ It has also been reported that an evolved HIV mutant (RT-1306) can directly read through m^1^A and generate mutations to allow m^1^A detection.^22^ More recently, we found that the Protoscript II (PSII) and SuperScript IV (SSIV) reverse transcriptase (RT) enzymes can read through the reduced form of m^1^A efficiently and generate high mutation rates.^23^ Based on these successful examples showing that some RT enzymes can read through modified bases and generate mutation signatures, we reasoned that a certain RT enzyme may be able to read through m^3^C and generate mutations to allow direct detection of m^3^C sites in biological samples.

We synthesized a series of RNA probes containing m^3^C, m^4^C, m^5^C, hm^5^C, and f^5^C, respectively (see Methods for probe sequences), and used them as substrates to screen HIV RT, RT-1306, and PSII to compare the mutation rates at the modified sites. Additionally, we expressed and purified AlkB and verified its demethylation activity by using it to treat a synthetic RNA oligo containing m^3^C. As shown in Figure 2A, Maldi TOF MS showed that the peak representing m^3^C at 2865 was quantitatively converted to 2851. The loss of 14 Daltons strongly supports the m^3^C demethylation activity of AlkB. It is worth noting that a small amount of peak at 2866 remained, but it likely represented the contamination of m^3^U which was generated in the deprotection step of synthesis of the RNA oligo containing m^3^C. The demethylation activity of AlkB was further verified by quantifying the m^1^A abundance from HeLa total RNA before and after AlkB treatment (Figure 2B).

With these materials on hand, we set up a screen by creating sequencing libraries of a mixture of total RNA and synthetic probes using either HIV RT, RT-1306, or PSII for reverse transcription. We additionally performed sequencing after AlkB demethylation using PSII. We found that the commercially available PSII performed better than WT HIV RT and the evolved mutant in terms of readthrough and mutation rates (Figure 2C). Use of PSII induced less RT stops before or after the m^3^C site compared to HIV RT and exhibited a slightly higher mutation rate than both HIV RT and RT-1306. For all the other cytosine modifications, only low levels of mutations were observed, suggesting that false positives caused from other cytosine modifications are low (Figure 2C).

Given these findings, we devised a method that uses PSII II and AlkB demethylation for m^3^C detection. Our method involves constructing two sets of libraries by splitting each sample into two parts and comparing the readout of m^3^C sites (see general method scheme in Figure 2D). One part is treated with AlkB to demethylate m^3^C so that the m^3^C site can be read as C in the RT reaction. The second part of RNA is split into technical replicates and the RT reaction is conducted using PSII without AlkB treatment. In this case, the m^3^C sites are expected to be read as U. Comparing the readouts of these two sets of libraries allows determination of m^3^C sites in sequencing results. This same library construction strategy has been successfully applied for m^1^A sequencing on mRNA by using the evolved HIV RT-1306 to read through m^1^A and generate mutations.^22^

Since m^3^C sites in tRNA and mt-tRNA have been reported^15^, we set up an experiment to validate our method by applying it to characterization of human m^3^C tRNA methylomes. We constructed untreated libraries using PSII in triplicate side by side with one AlkB-treated library as a control starting from the small RNA fraction (<200 nt) of HeLa. To further investigate whether the known m^3^C sites in tRNA and mt-tRNA are METTL8 substrates, we also constructed the corresponding libraries with small RNA fractions purified from cells with *METTL8* knockdown or overexpression.

Table 1 shows all the m^3^C sites in tRNA and mt-tRNA detected by our method and a comparison with all the m^3^C sites reported by selected other methods.^15,16,24^ We were able to detect 17 m^3^C sites in tRNA, 16 of them were also detected by HAC-seq and DM-tRNA-seq. One site was detected by our method but not by the other methods listed. One site was identified by HAC-seq and DM-tRNA-seq but not detected in our results, and another was detected by HAMR but not by the other methods listed, including ours. For all the m^3^C sites detected by our method, mutation rates were substantially decreased with AlkB treatment (Figure 3 and Supplementary Table 1). We compared the mutation rates at the m^3^C sites detected in different libraries involving *METTL8* KD or OE to determine whether METTL8 may be responsible for their installation. We found that none of the m^3^C fractions in the cytosolic tRNAs were sensitive to *METTL8* KD or OE (Figure 3 and Supplementary Table 1), suggesting that these m^3^C sites are not METTL8 substrates. In contrast, our data showed that the mt-tRNA^Thr(TGT)^ and mt-tRNA^Ser(TGA)^ m^3^C sites were sensitive to both *METTL8* KD and OE. For example, the m^3^C mutation rate at mt-tRNA^Thr(TGT)^ was determined to be ~79% on average in three replicates of the untreated libraries while the AlkB-treated library showed no mutation. In the *METTL8* KD libraries, the mutation rates decreased to ~27% on average in the three replicates. Accordingly, in the *METTL8* OE libraries, the mutation rates increased from ~75% in the overexpression control to ~84% in OE samples (Figure 3, Figure 4A–B, and Supplementary Table 1), with a dominant mutation pattern of C-to-U. The mt-tRNA^SerTGA^ m^3^C site also showed the same pattern for *METTL8* knockdown (Figure 3, Figure 4C–D and Supplementary Table 1). These results confirmed that the mt-tRNA^Thr(TGT)^ and mt-tRNA^Ser(TGA)^ m^3^C sites are installed by METTL8 and validated that our method can detect the m^3^C modification and its dynamics in biological samples with high sensitivity and accuracy. Additionally, we detected a candidate m^3^C site in tRNA-Ala^(AGC)^ at position C24 with a mutation rate of ~34% in untreated libraries and with the AlkB-treated library showing a decrease in mutation rate to ~2% at this position. Mutation levels at this position in untreated *METTL8* KD and *METTL8* OE samples were ~29% and 27%, respectively, suggesting METTL8 is likely not the m^3^C writer for this candidate site (Figure 3, Figure 4E–F, and Supplementary Table 1).

After validating our method by successfully detecting m^3^C in the spike-in oligos, tRNA, and mt-tRNA, we then applied it in a pilot study to map m^3^C sites in mRNA. We purified polyA-enriched RNA from stable cell lines with either non-targeting control or *METTL8*-targeting shRNA and constructed two input libraries and one AlkB-treated library. Knockdown efficiency of *METTL8* was validated by qPCR (Supplementary Figure 1A). Sequencing data from our pilot study showed mutations at numerous C sites that decreased with AlkB treatment. When applying an analytical pipeline involving stringent filtering for sites with at least 20 reads, an average mutation rate of at least 0.2, and at least 50% demethylation by AlkB, we identified 4 candidate poly(A)^+^ RNA m^3^C sites (Supplementary Table 3). Of the sites identified, we noted that SLC25A6 (also known as ADT3) displayed high mutation rates in both control HeLa input samples (i.e., m^3^C presence) that were significantly decreased in the AlkB sample and both *METTL8* knockdown samples (NB: SLC25A6 is on the minus (−) strand, so the RNA m^3^C→A mutation is displayed as G→T in IGV). Additionally, a motif for this putative m^3^C site resembles the motif in the two known m^3^C sites in mt-tRNAs (m^3^CUU) (Supplementary Figure 1B). Candidate sites revealed in this study would need to be confirmed with future biological studies involving additional replicates and deeper sequencing which would allow more robust statistical analyses, for which this pilot study lays important groundwork.

## DISCUSSION

Our studies have revealed that PSII has high readthrough and mutation rates for untreated m^3^C sites (Figure 2C), better than HIV RT which has previously been applied for this purpose. Therefore, we devised a method which utilizes PSII in combination with AlkB demethylation to detect m^3^C in RNA at single-base resolution.

We applied our method to sequence small RNA from human cell lines and were able to successfully identify known m^3^C sites in tRNA and mt-tRNA (Figures 3–4). We additionally detected a candidate m^3^C site in tRNA^Ala(AGC)^ at position C24, which had lower mutation rates than most of the other m^3^C sites we detected in tRNA, but which was still sensitive to AlkB demethylation (Figure 4E–F). To the best of our knowledge, this has not previously been identified as a putative m^3^C site. C24 in tRNA^Ala(AGC)^ base pairs with G10 in models of tRNA^Ala(AGC)^ secondary structure (CITE); m^3^C methylation at this position would likely disrupt this base pairing with possible impacts on tRNA structure and stability. Therefore, further investigation of this site could uncover interesting functional roles. Furthermore, our method was also sensitive to changes in methylation level at METTL8-responsive m^3^C sites in mt-tRNA upon METTL8 depletion and overexpression (Figure 3, Figure 4 A–D). Therefore, we envision that our method could be applied for future studies of METTL8 or other tRNA m^3^C effector proteins.

Pilot studies with poly(A)^+^ RNA showed that libraries could be prepared without significant degradation due to the lack of harsh steps included in some other methods, and a few candidate m^3^C sites were identified through these preliminary studies (Table S3, Figure S1B). All identified sites in poly(A)^+^ RNA had much lower mutation rates than most tRNA m^3^C sites. The abundance of m^3^C in poly(A)^+^ RNA is much lower than in small RNA (Figure 1B), so it is expected that fewer sites of high stoichiometry would be detected. Our data suggest that the m^3^C detected in poly(A)^+^ RNA by mass spectrometry is likely coming from numerous m^3^C sites of low stoichiometry spread across many transcripts, in line with HAC-seq data which did not reveal any m^3^C sites in mRNA at comparable levels to those in tRNA.^15^ It is worth mentioning that low overlap in identified sites existed between the two input replicates in poly(A)^+^ RNA libraries, and we often observed high variation in mutation rates and AlkB demethylation rates between replicates, even for the same sites. These results emphasize that further optimization should be explored, potentially including condition optimization and adjustments to the bioinformatic analysis pipelines. Future endeavors beyond these pilot experiments should also include additional biological and technical replicates to measure robustness and reproducibility, and to increase confidence in identified sites.

Despite these limitations, our method was robust in its ability to identify known m^3^C sites on probes as well as in tRNA and mt-tRNA (Figure 2C, Figures 3–4), and its application to human RNA samples provided several interesting insights that hint at avenues of future investigation. Given that PSII is commercially available, and the method design is straightforward, it could easily be applied to future functional studies to identify additional tRNA m^3^C effector proteins or study the roles of these known m^3^C sites in cells under various conditions. Therefore, we envision that future work could build off of our findings to study m^3^C function in RNA biology.

## MATERIALS AND METHODS

### Cell culture and transfection

Hela cells and derived stable clones were maintained in RPMI supplemented with 10% FBS as previously described.^27^ For lentivirus production, lentiviral constructs TRCN0000236515 and SHC002 (pLKO.1-derived plasmid with target sequence GATCGCCGCTTACAAGTTAAT for *METTL8* and CAACAAGATGAAGAGCACCAA as control) together with pCMV-dR8.2 dvpr and pCMV-VSVG were co-transfected into 293TN cells (System Biosciences). Viruses were concentrated using PEG-it Virus precipitation solution (System Biosciences) and used for infecting cells in the presence of TransDux (System Biosciences). Pools of stable transfectants were selected with 2μg/ml puromycin.

### RNA purification

Total RNA was isolated from HeLa cells using Trizol (Invitrogen) according to the manufacturer’s instructions. Polyadenylated RNA was isolated from total RNA using the Dynabeads mRNA Direct kit (Life Technologies) as per manufacturer instructions. Chromatin-associated RNA was purified as previously described by Liu *et al*.^28^ Small RNA was isolated from total RNA using the RNA Clean and Concentrator kit (Zymo) according to the manufacturer protocol for purification of small and large RNAs into separate fractions, saving the small RNA fraction.

### Synthesis and purification of RNA probes

RNA oligonucleotides used in this study were synthesized in-house using an Expedite DNA synthesizer, followed by normal deprotection for regular oligonucleotides. After deprotection, the RNA oligonucleotides were purified through HPLC with a C18 column and were eluted with 0–20% acetonitrile in 0.1 M triethylammonium acetate. The desired peak was collected and dried by lyophilization.

### HPLC oligonucleotide purification

The deprotected crude mixture of RNA probes was loaded onto a C18 reverse column (Higgins Analytical, Inc.) using Waters e2695. Peaks were collected and dried using SpeedVac Vacuum Concentrator.

### PAGE-gel oligonucleotide purification

5–10 μg of RNA probe was mixed with 2x RNA loading buffer (9M urea, 100 mM ETDA (pH 8.0), 0.2% (w/v) bromophenol blue) and loaded onto 15% acrylamide/bis solution 29:1 (Bio-Rad Laboratories), 7M Urea, 1x TBE buffer PAGE gel. The gel was run at 18W for ~2 hours. RNA bands were excised from the gel by visualizing with a UV lamp. Gel pieces were crushed with a pipette tip and eluted in gel elution buffer (200 mM KCl, 50 mM KOAc, (pH 7.0)) overnight at 4 °C on a hula mixer. Next, the samples were spun at 4 °C for 30 min. at 21,000 g and RNA was purified from the supernatant by ethanol precipitation.

### Quantitative PCR

Quantitative PCR was performed as previously reported.^29^ The primers used to target *METTL8* had the following sequences: TGCGAGTCCTTCTGGAAGAGCA (forward) and CTCAACAGCCAATTACGATCCTTG (reverse).

### Mass spectrometry

LC-MS/MS was performed as previously reported,^29^ with a few key differences. For liquid chromatography, samples were separated on a HypersilGold aQ50 C18 50×3mm column. C and m^3^C nucleosides were quantified using the mass transitions 258.2>126.1 (m^3^C) and 244>112 (C).

For MALDI-time-of-flight (MALDI-TOF) and HPLC, probes from AlkB demethylation reactions were purified by ethanol precipitation. Samples were then mixed with MALDI matrix, spotted and dried on a MALDI sample plate, and analyzed by a Bruker Ultraflextreme MALDI-TOF-TOF mass spectrometer in a reflector, positive mode similarly to a previous report.^30^

### AlkB expression, purification, and activity testing

AlkB was overexpressed using a vector from Addgene (#79050) in *E. coli* BL21 (DE3) following previously reported protocols.^21^ Cells were induced to overexpress AlkB by adding IPTG to a final concentration of 1 mM and iron (ferrous) sulfate to a final concentration of 20 μM. After shaking at 16 °C overnight, the culture was pelleted and resuspended in lysis buffer (20 mM Tris-HCl pH 7.5, 300 mM NaCl, 5 mM imidazole, 1x EDTA-free protease inhibitor (Roche)). The recombinant AlkB protein was purified using a Ni-NTA super-flow column (QIAGEN). Fractions were checked for the presence and purity of AlkB by running them on a 4–12% Bis-Tris PAGE gel in 1x MOPS buffer and staining with Coomassie blue. Fractions with strong signals for AlkB at ~25 kD and low background contaminants were pooled and stored at −80 °C for future use. The AlkB demethylation activity assay was performed as described by Zhang *et al*.^17^

### m^3^C-seq

#### Preparation of libraries for initial screen:

Initial libraries were prepared from RNA samples purified from wild type HeLa cells. The screen in Figure 2C was performed beginning with a mixture of total RNA (0.5 μg) and small RNA (1.5 μg). RNA was fragmented with 10x fragmentation reagent (100 mM ZnCl_2_, 10 mM Tris pH 7.5) at 70 °C for 10 minutes and purified using the RNA Clean and Concentrator kit (Zymo), eluting with 45 μL of RNase-free water. 3′-repair was conducted by adding 5 μL T4 PNK 10x buffer and 1 μL T4 PNK (NEB) followed by incubation at 37 °C for 1 hour, purification with the Oligo Clean and Concentrator kit (Zymo) eluting with 13 μL RNase-free water, and measurement of the concentration. 100 ng was then taken for 3′-ligation, along with 2 ng of spike-in oligo mixture with sequences indicated at the end of this section. 1 μL of 11.25 μM 3′-adaptor (5′-rApp-NN NNN ATC ACG AG ATC GGA AGA GCA CAC-3′) was added to this mixture, and it was heated at 70 °C for 2 minutes and put onto ice. Then, 10 μL 3′-ligation buffer and 3 μL ligation enzyme mix were added (from the NEBNext Multiplex Small RNA Library Prep Set for Illumina). After pipetting, the reactions were incubated at 16 °C for 16 hours. Digestion of excess 3’-adaptor was then performed by adding 28 μL water and 1 μL 5’-deadenylase (NEB #M0331) into the ligation mix and incubating at 30 °C for 1 hour, followed by adding 1 μL RecJF I (NEB) at 37 °C for 1 hour and purification by RNA Clean and Concentrator kit (Zymo) with 120 μL ethanol and elution by 31 μL RNase-free water. The sample was then split to test different reverse transcriptases and conditions. To test each RT, 2 μL of each sample was combined with 1 μL 1.125 μM SR RT primer (NEBNext Small RNA kit) and incubated at 65 °C for 5 minutes then placed on ice. Then, 4 μL of 5x buffer (HIV RT buffer (250 mM Tris-HCl (pH 8.3), 375 mM KCl, 10 mM MgCl_2_) or 5x ProtoScript^®^ II Reverse Transcriptase Reaction Buffer (NEB), 2 μL 10 mM dNTP, and 2 μL of each RT were added with water to 20 μL. The mixture was incubated at 37 °C (HIVRT and HIVRT-1306) or 42 °C (PSII) for 1 hour. 1 μL murine RNase inhibitor was included in the PSII library. 8 μL of 3′-ligated sample was used for AlkB treatment followed by sequencing with PSII. AlkB treatment was performed as described previously.^17^ Briefly, RNA was treated in a 50-μL reaction volume with the following reagents: 30 mM MES buffer (pH 5.5), 100 μM (NH_4_)_2_Fe(SO_4_)_2_·6H_2_O, 300 μM α-ketoglutarate, 2 mM L-ascorbic acid, 150 mM NaCl, 2 mM MgCl_2_ and 40 μg ml^−1^ BSA with 2.5 μl SUPERase•In RNase Inhibitor and 1.0 μl of engineered AlkB^16,17^ (D135S, 10 mg ml^−1^). The mixture was incubated at 25 °C for 2 h and then purified using the Oligo Clean & Concentrator kit (Zymo). 100 ng 90-mer polyT DNA carrier (IDT) was added to the sample and it was purified with the Oligo Clean and Concentrator kit (Zymo). 9 μL was then taken for reverse transcription with PSII similarly to the reaction described above. After the RT reactions, 1 μL RNase H buffer and 1 μL RNase H (NEB) were added to each reaction, with incubation at 37 °C for 30 minutes. 28 μL RNase free water was added followed by purification with the RNA Clean and Concentrator kit (Zymo), using 120 μL ethanol and eluting with 6.5 μL RNase-free water. Next, 3’ cDNA ligation was performed by adding 1 μL 50 μM 3′ cDNA linker (5’-/Phos/NNN NNG ATC GTC GGA CTG TAG AAC TCT GAAC /3SpC3) and 1 μL DMSO into 5.5 μL of each cDNA sample. Samples were heated at 70 °C for 2 minutes, chilled on ice immediately, and then a ligation mixture was added consisting of 2 μL 10x RNA ligation buffer (NEB), 0.2 μL 100 mM ATP, 9 μL 50% PEG8000, and 1.5 μL Rnl1 (NEB M0437M, high concentration). Ligation was performed at 25 °C for 12 hours, followed by incubation at 65 °C for 5 minutes and a hold at 4 °C. Purification was performed using the RNA Clean and Concentrator kit (Zymo), using 100 μL ethanol and eluting with 20 μL RNase-free water. qPCR was then conducted to test the cycles needed for library amplification 10 μL FastStart Essential DNA Green Master Mix (Roche), 1 μL index 1 primer (NEB small RNA kit), 1 μL SR primer (NEB), 1 μL cDNA template, and 7 μL RNase-free water on a LightCycler96 (Roche). PCR was performed for each sample using the appropriate number of cycles per sample by combining 12.5 μL 2x LongAmp Taq Mix (NEB small RNA kit), 0.625 μL SR primer (NEB small RNA kit), 1.25 μL RNase-free water, 10 μL cDNA, and 0.625 μL of an index primer unique to each sample (NEB). Samples were pre-incubated at 94°C for 30s, followed by cycling for a number of cycles determined by qPCR at 94°C for 15 seconds, 62 °C for 30 seconds, and 70 °C for 15 seconds, and finishing with a 70°C, 5-minute final extension. Libraries were purified using a 3% low melting agarose gel using 6x loading buffer (NEB #B7024) and the Quick Load pBR322 DNA-MspI Digest NEB #N3032 ladder. Samples were run at 90 V for 1–1.5 hours and purified by MinElute Gel Extraction Kit (Qiagen). Samples were sequenced on a Novaseq6000 SP flowcell with 0.88 Billion reads.

#### Spike-in oligo sequences used in initial screen:

**DH-718–5-33mer:** 5’-GGA GAC GGU CGG Gm^3^CC CAG AUA UUC GUA UCU GUC-3’
**DJ-963–11-45mer:** CCU CAC CAU CUC AAC CUG AGU CG(5mC) GGC AUG AGG GAG AAG UGG UGA
**m**^**4**^**C oligo**: GGA ATA AAA TGA ATA (m^4^C)AG AAT TAA GGA GGA ATA AAA TAT TAG AGG AAT AAA ATA TTA AGG
**DO-12–16a-21mer:** 5′-ACU CAGATC UCG UG(hm^5^C) UAC GCU
**DJ-963–13-27mer:** 5′-UGU CAU CAC GAG G(f^5^C)A CUA AAG CUG ACU

Libraries for small RNA from *HeLa shCtrl, shMETTL8, OE Ctrl and METTL8 OE* were created similarly to the procedure outlined above, with some modifications. RT was performed only with PSII. Each sample was split into four parts, with one part undergoing AlkB treatment, such that there were three technical replicates for the untreated condition and one for the AlkB condition. Spike-in probes included only two m^3^C containing probes, with the following sequences:

**DP-518–6-27mer:** 5′-UAC ACU CAG m^3^CUC GGA CUA AAG CUG −3′
**DH-718–5-33mer:** 5′-GGA GAC GGU CGG Gm^3^C CAG AUA UUC GUA UCU GUC-3′

#### Preparation of HeLa shCtrl, shMETTL8, OE Ctrl and METTL8 OE small RNA sequencing libraries:

300 ng of small RNA was mixed with 3 μL of 1 ng/μL mixture of spike-in m^3^C probes and split into two for 3’ RNA ligation. RNA in 9 μL RNase-free water was mixed with 1 μL of 11.25 μM 3′-adaptor (5-’rApp-NN NNN ATC ACG AG ATC GGA AGA GCA CAC-3’) and incubated at 70 °C for 2 minutes. Then, after chilling on ice for 2 minutes, 2 μL of RNase-free water, 2.5 μL of 10x T4Rnl1 buffer (NEB #B0216S), 7.5 μL of 50% PEG800 (NEB #B1004S), 1 μL of SuperaseIn (Invitrogen, AM2694) and 2 μL of T4 RNA ligase 1 truncated KQ (NEB #0373) were added and mixed well. The reaction mixture was incubated at 25 °C 1 h followed by 16 °C for 12 h. Then, RNase-free water was added to the total volume of 47 μL and 2 μL of 5′-deadenylase (NEB #M0331) was added, mixed and incubated at 30 °C for 1 h. Next, 1 μL of RecJf (NEB, M0264S) was added, mixed and incubated at 37 °C for 1 h. After that, two ligation mixtures coming from the same sample were pooled together, purified by RNA Clean and Concentrator kit (Zymo) and eluted with 31 μL RNase-free water. Then, sample was split into four parts: 3 × 3 μL were used as three technical replicates and 20 μL was treated with AlkB as described previously.^17^ After treatment with AlkB, reverse transcription was performed as follows: To RNA in 8.5 μL RNase-free water, 1 μL of 3.75 μM (input) or 1 μL of 11.25 μM (AlkB-treated) SR RT primer (5′-AGA CGT GTG CTC TTC CGA TCT, IDT) was added and incubated at 65 °C for 5 minutes then chilled on ice for 2 min. Then, 4 μL of 5x PSII buffer, 2 μL of 0.1 M DTT, 2 μL of 10 mM dNTPs, 0.5 μL of RNase inhibitor (NEB, M0314) and 1 μL of PSII (NEB M0368L) were added, mixed and incubated at 42 °C for 1h followed by overnight incubation at 35 °C for 16 hours. Next, 1 μL of RNase H buffer and 1 μL of RNase H were added, mixed and incubated at 37 °C for 30 min. Then, sample volume was adjusted to 50 μL with Nuclease-free water and purified by RNA Clean and Concentrator kit (Zymo) and eluted with 6.5 μL of Nuclease-free water. cDNA ligation was performed as follows: 1 μL of 10 μM 5’ linker (5′-/Phos/NNN NNG ATC GTC GGA CTG TAG AAC TCT GAA C/3SpC3, IDT) and 1 μL of DMSO were mixed with 5.5 μL of cDNA and incubated at 70 °C for 2 min. and then chilled on ice for 2 min. Next, 2 μL of 10x RNA ligation buffer (NEB #B0216S), 0.2 μL of 100 mM ATP, 9 μL of 50% PEG8000 (NEB, B1004S), 0.6 μL of 50 mM hexaamminecobalt (III) chloride [Co(NH_3_)_6_Cl_3_] (Sigma-Aldrich 481521), 0.4 μL of 5’-deadenylase (NEB, M0331) and 1.5 μL of T4Rnl1 high concentration (NEB, M0437M) were mixed and incubated at 25 °C for 16 hours. Then, 7 volumes of DCC binding buffer were added and samples were purified by DNA Clean and Concentrator kit (Zymo) and eluted with 20 μL Nuclease-free water. qPCR was then conducted to test the cycles needed for library amplification: 10 μL FastStart Essential DNA Green Master Mix (Roche), 1 μL index 1 primer (NEB small RNA kit, E7330S), 1 μL SR primer (NEB, E7330S), 1 μL cDNA template, and 7 μL RNase-free water on a LightCycler96 (Roche). PCR was performed for each sample using the appropriate number of cycles per sample by combining 12.5 μL 2x LongAmp Taq Mix (NEB small RNA kit), 0.625 μL SR primer (NEB small RNA kit), 1.25 μL RNase-free water, 10 μL cDNA, and 0.625 μL of an index primer unique to each sample (NEB). Samples were pre-incubated at 94 °C for 30s, followed by cycling for a number of cycles determined by qPCR at 94 °C for 15 seconds, 62 °C for 30 seconds, and 70 °C for 15 seconds, and finishing with a 70 °C, 5-minute final extension. Libraries were purified using a 3% low melting agarose gel using 6x loading buffer (NEB #B7024) and the Quick Load pBR322 DNA-MspI Digest NEB #N3032 ladder. Samples were run at 90 V for 1–1.5 hours and purified by MinElute Gel Extraction Kit (Qiagen). Samples were sequenced on the NextSeq2000 system (Illumina).

#### Preparation of HeLa sh-control and sh-METTL8 poly(A)^+^ and caRNA libraries:

For sequencing of HeLa sh-control and sh-METTL8 libraries, 200 ng of each RNA sample was brought to 18 μL with RNase-free water. Then 2 μL 10x fragmentation reagent (100 mM ZnCl_2_, 10 mM Tris pH 7.5) was added to each sample, followed by incubation at 70°C for 5 minutes and placement on ice for 1 minute. Then, 0.5 M EDTA pH 8 was added to each sample, followed by purification using the Oligo Clean and Concentrator kit (Zymo) with a 1:2:8 ratio of sample to binding buffer to ethanol. 3’ repair was performed by adding 2 μL of 10x PNK buffer and 1 μL T4 PNK (NEB) to 17 μL RNA sample, incubating at 37 °C for 1 hour. Then, samples were purified using the RNA Clean and Concentrator kit (Zymo) using 40 μL binding buffer, 32 μL ethanol, 160 μL prep buffer, and 700 μL wash buffer, followed by elution with 7.5 μL of RNase-free water. 3’ ligation was initiated by combining 6 μL of 3′-repaired RNA with 1 μL of 5.625 μM 3’-adaptor (5-′-rApp-NN NNN ATC ACG AG ATC GGA AGA GCA CAC-3′) and heating the mixture at 70 °C for 2 minutes, then placing on ice 1 minute. 10 μL 3′-ligation buffer and 3 μL enzyme mix (from the NEBNext Multiplex Small RNA Library Prep Set for Illumina) were added, and the mixture was incubated at 16 °C for 16 hours. 1 μL 5’ deadenylase (NEB #M0331) was added followed by incubation at 30 °C for 1 hour, then 1 μL RecJF (NEB) was added followed by incubation at 30°C for 1 hour. Samples were purified with the RNA Clean and Concentrator kit (Zymo) using 44 μL binding buffer, 30.8 μL ethanol, 176 μL prep buffer, 700 μL wash buffer, and 9.5 μL RNase-free water for elution. About ⅛ of the sample was aliquoted into each of two new tubes to create two input samples. The rest was subjected to AlkB treatment and filled up to 20 μL with RNase-free water. AlkB treatment was performed as previously reported.^17^ Samples of 50 μL were then purified using the Oligo Clean and Concentrator kit (Zymo) using 100 μL binding buffer, 400 μL ethanol, 750 μL wash buffer, and 11 μL RNase-free water for elution. Reverse transcription was then initiated by combining 9.5 μL RNA sample in water with 1 μL 1.25 μM SR RT primer (NEBNext Small RNA kit) and incubating at 65 °C for 5 minutes, then placing on ice for 2 minutes. To each sample, 4 μL of 5x ProtoScript II buffer, 2 μL 0.1M DTT, 2 μL 10 μM dNTPs, 0.5 μL RNase inhibitor, and 1 μL ProtoScript II (NEB) were added and mixed, and the reaction was incubated at 42 °C for 1 hour. After the reverse transcription reaction, 1 μL of RNase H buffer and 1 μL of RNase H (NEB) were added followed by incubation at 37°C for 30 minutes. Purification of samples was performed using the RNA Clean and Concentrator kit with 44 μL binding buffer, 44 μL ethanol, 176 μL prep buffer, and 700 μL wash buffer. Then, the 3’ cDNA linker (5’-/Phos/NNN NNG ATC GTC GGA CTG TAG AAC TCT GAAC /3SpC3) was ligated by mixing 1 μL 50 μM linker and 1 μL DMSO with 5.5 μL cDNA sample and incubating at 70 °C for 2 minutes followed by placement on ice for a few minutes during preparation of the ligation mixture. The ligation mixture for each sample included 2 μL 10x RNA ligation buffer (NEB), 100 mM ATP, 50% PEG8000, and 1.5 μL T4 RNA ligase 1 (NEB M0437M, high concentration). Importantly, all reagents except T4 RNA ligase 1 were prepared as a master mix before aliquoting to samples, and T4 RNA ligase 1 was added directly to each sample with immediate mixing. Ligation was performed at 25 °C for 12 hours, followed by heat inactivation at 65 °C for 5 minutes. Samples (20 μL each) were purified using the RNA Clean and Concentrator kit (Zymo) by using 40 μL binding buffer, 24 μL ethanol, 160 μL prep buffer, 700 μL wash buffer, and 21 μL RNase-free water for elution. qPCR reactions to test cycle numbers were set up with 10 μL FastStart Essential DNA Green Master Mix (Roche), 1 μL index 1 primer (NEB small RNA kit), 1 μL Universal primer (NEB), 1 μL cDNA template, and 7 μL RNase-free water. qPCR was performed as described above. PCR was performed for each sample using the appropriate number of cycles per sample by combining 12.5 μL 2x LongAmp Taq Mix (NEB small RNA kit), 0.625 μL SR primer (NEB small RNA kit), 1.25 μL RNase-free water, 10 μL cDNA, and 0.625 μL of an index primer unique to each sample (NEB). Samples were pre-incubated at 94 °C for 30s, followed by cycling for a number of cycles determined by qPCR at 94 °C for 15 seconds, 62 °C for 30 seconds, and 70°C for 15 seconds, and finishing with a 70 °C, 5-minute final extension. Libraries were purified by AMPure bead purification (Beckman Coulter) per manufacturer instructions. Sample concentrations were measured by Qubit and quality control analysis was performed by Bioanalyzer. Libraries were sequenced on a NovaSeq6000 SP flow cell twice, once with 100bp single end reads and again with 50bp paired end reads (due to a technical processing error at the sequencing facility).

#### Data analysis for initial screen:

Reads were trimmed using the trim command in HomerTools^31^, and low-quality reads were subsequently removed. The remaining high-quality reads were then mapped to spike-in sequences using BWA^32^ with the following parameters: ‘bwa aln -t 4 -n 0.06 -q 20’ for downstream analysis.

#### Data analysis for HeLa sh-Control and sh-METTL8 poly(A)^+^ and caRNA libraries:

Read1 files from both sequencing experiments were merged using the Linux command cat and then quality checked using FastQC v0.11.5 (http://www.bioinformatics.babraham.ac.uk/projects/fastqc/). Then, analysis was performed using a pipeline constructed by modifying the framework used by Zhou *et al*.^22^ Unique molecular identifiers (UMIs) were extracted using UMI-tools.^33^ Adapters were trimmed using BBDuk from BBTools version 38.84 and mapped to human genome hg38^34^ using hisat2 v2.1.0.^35^ Sorting of strands was performed using samtools view with options -F 0×10 for positive strand reads and -f 0×10 for negative strand reads, followed by sorting and indexing with the samtools sort and index commands.^36^ Deduplication was then performed with UMI-tools.^33^ To count the number of reads with each base at each position, we employed bam-readcount (https://github.com/genome/bam-readcount). To count mutation rates at each base that is C in the reference genome, apply total read cutoff and mutation cutoff filters, and clean site lists, we used versions of the python scripts “parse.py”, “control_parse.py”, and “clean_untreat.py” from Zhou *et al*. modified to use C as a reference base for positive files and G for negative files.^22^ We then used R v. 4.0.3 (https://cran.r-project.org/) for downstream analyses, beginning with versions of the scripts “Prepare.R” and “Callm1A-input.R” modified for analysis of m^3^C.^22^ Cutoffs for read counts, mutation rate, and demethylation rate are indicated in the text and figure captions in relevant sections. Putative m^3^C sites were spot-checked by visualization using Integrated Genomics Viewer (https://software.broadinstitute.org/software/igv/). Plots were created using R v. 4.0.3 (https://cran.r-project.org/).

## Supplementary Material

FigS1

Supplementary Table 1 **Table S1.** Mutation rates at m^3^C sites in human tRNA and mt-tRNA in *METTL8* KD and *METTL8* OE libraries compared to the appropriate controls.

Supplementary Table 2 **Table S2.** Coverage and mutation data for modification sites on each probe under each condition included in the RT screen (HIVRT, HIVRT-1306, PSII, and PSII + AlkB).

Supplementary Table 3_260619 **Table S3.** Coverage, mutation rate, demethylation rate, and annotation data for m^3^C sites identified in both replicates of poly(A)^+^ RNA input libraries with mutation rates that decrease by at least 50% with AlkB treatment.

## Figures and Tables

**Figure 1.**
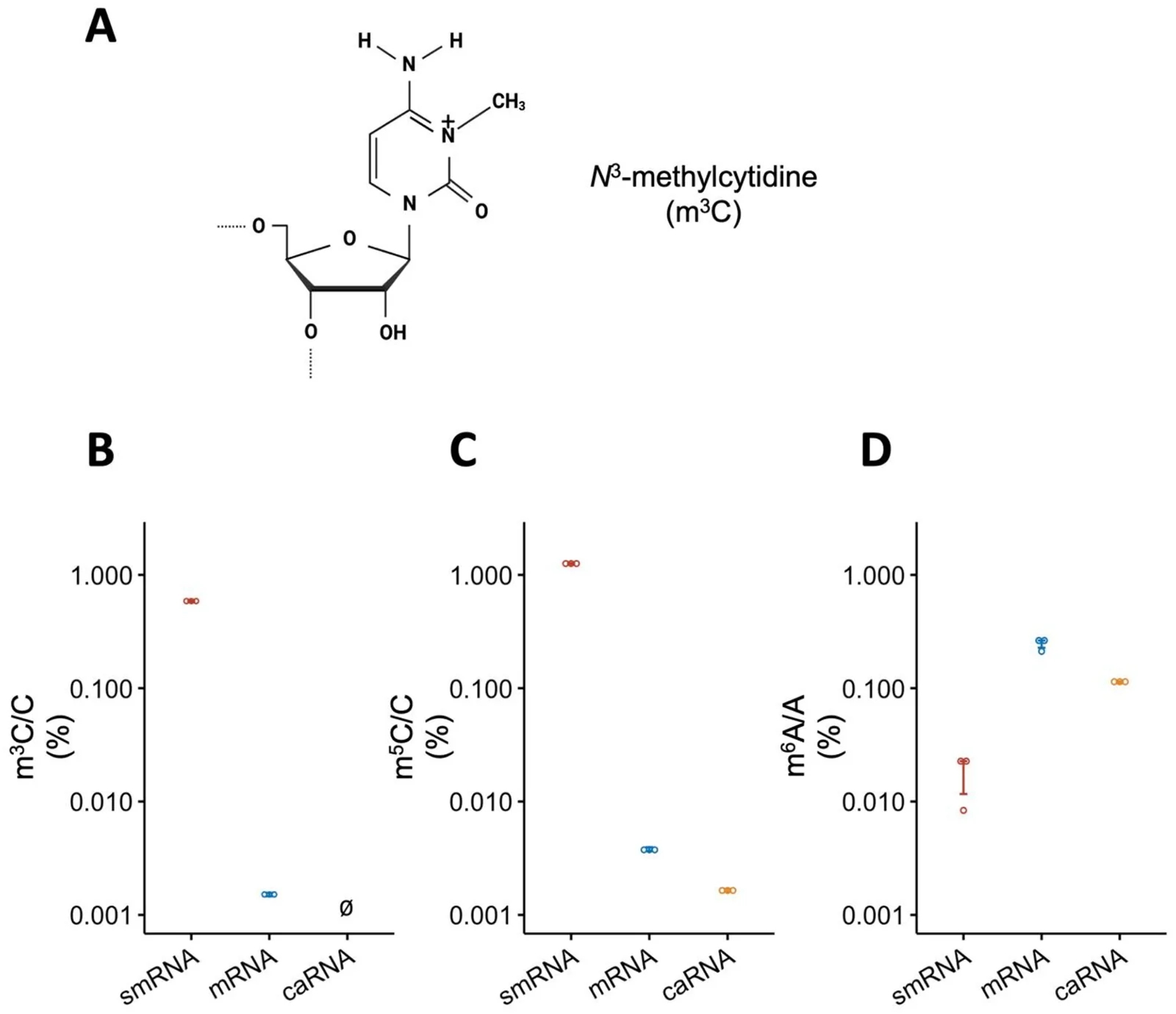
*N*^3^-methylcytidine in human RNA. (**A**) The chemical structure of *N*^3^-methylcytidine (m^3^C). (**B-D**) LC-MS/MS analysis of m^3^C (B), m^5^C (C), and m^6^A (D) levels normalized to cytidine (B and C) or adenine (A) from different RNA types isolated from HeLa cells, shown using a log_10_ scale. smRNA = small RNA, mRNA = messenger RNA, caRNA = chromatin-associated RNA. Mean and s.e.m. for n=3 biological replicates were plotted.

**Figure 2.**
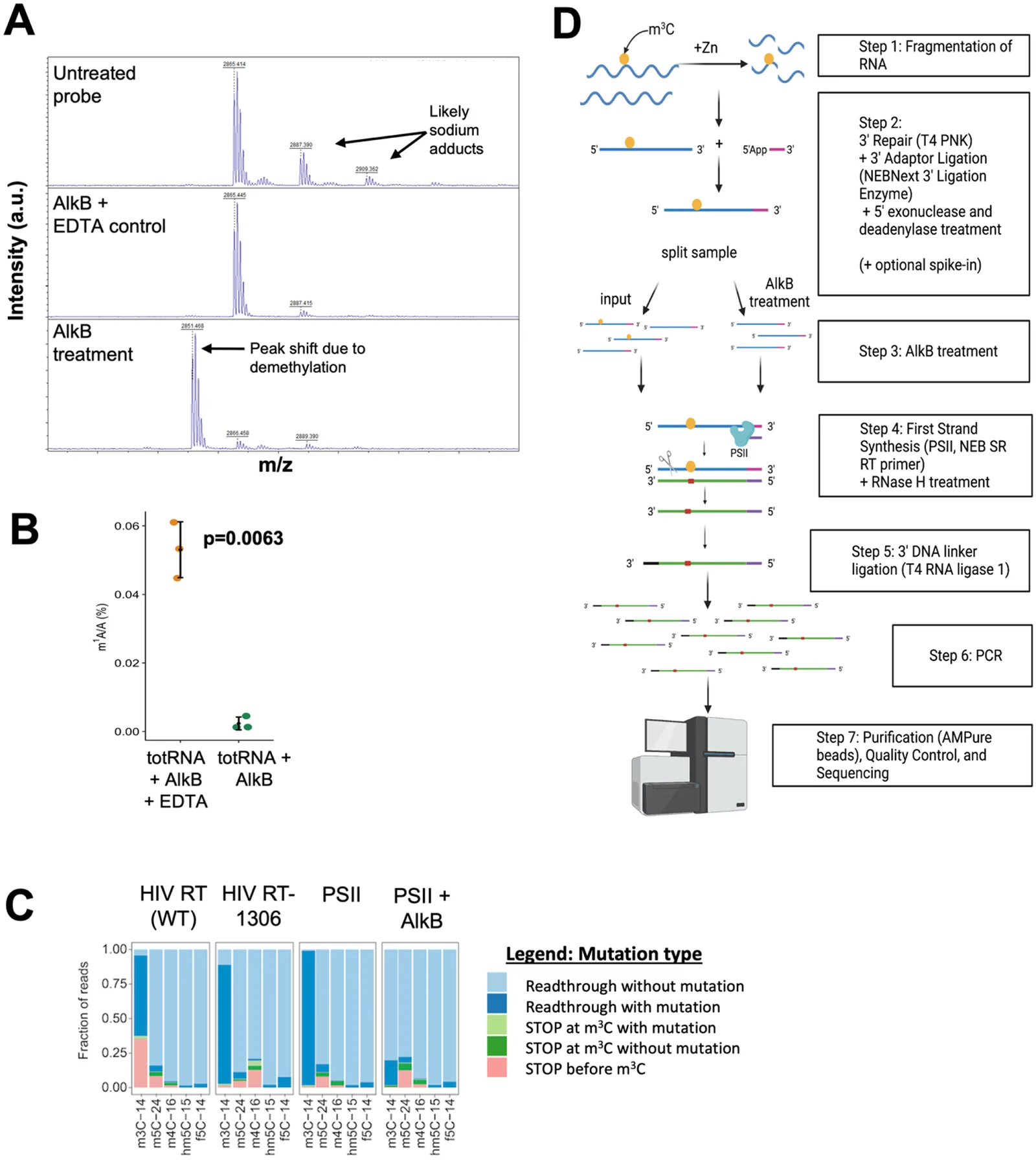
Method development for sequencing *N*^3^-methylcytidine in human RNA. (A) Intensity versus mass-to-charge ratio from matrix-assisted laser desorption ionization (MALDI) characterization of m^3^C-containing probes from three conditions: untreated, AlkB treated with EDTA to chelate iron, and AlkB treated. A shift in the major peak of −15 is shown in the AlkB treated sample, indicating loss of a methyl group. (B) LC-MS/MS analysis of m^1^A levels normalized to adenosine (A) from *in vitro* demethylation reactions of AlkB on HeLa total RNA are shown with (left) or without (right) EDTA. n=3 replicates. Mean and s.e.m. were plotted. *P* value was calculated using a two-sided T-test. (C) Fractions of reads with different mutation types at modification sites on probes from reverse transcription reactions using three different reverse transcriptases, HIV RT, HIV RT-1306 (evolved for m^1^A detection), and PSII, as well as from a reaction with PSII after AlkB treatment. See Methods section for probe sequences. Mutation types are indicated by color (light blue = readthrough without mutation, dark blue = readthrough with mutation, light green = stop after m^3^C site with a mutation, dark green = stop after m^3^C site without a mutation, pink = stop before the m^3^C site). (D) A schematic of the library preparation method employed for m^3^C-sequencing in this study.

**Figure 3.**
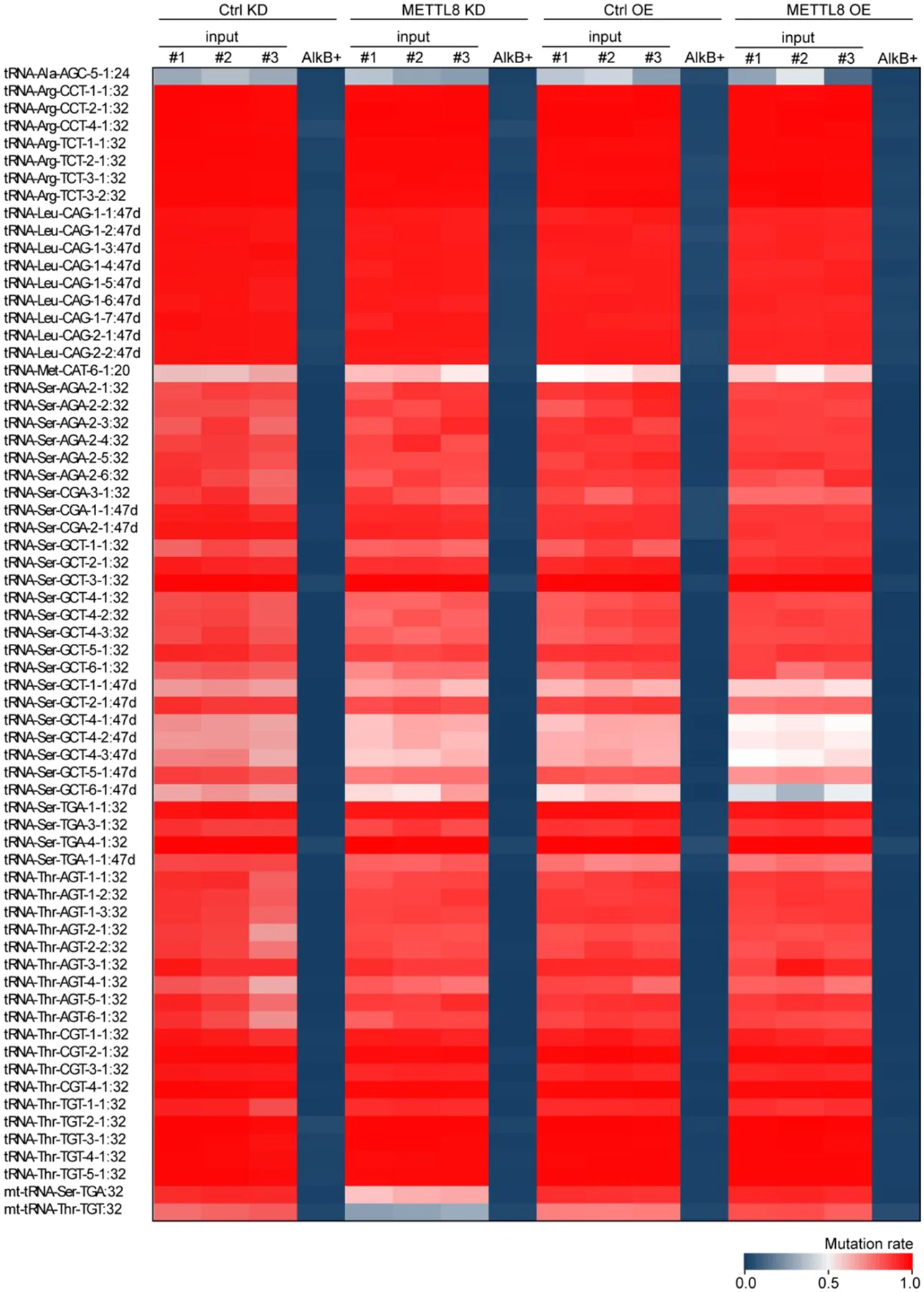
m^3^C sites in human tRNA. Heatmap showing the average mutation rates of detected m^3^C sites in cytosolic and mitochondrial tRNAs in input and AlkB-treated HeLa *METTL8* KD and OE samples and appropriate control samples.

**Figure 4.**
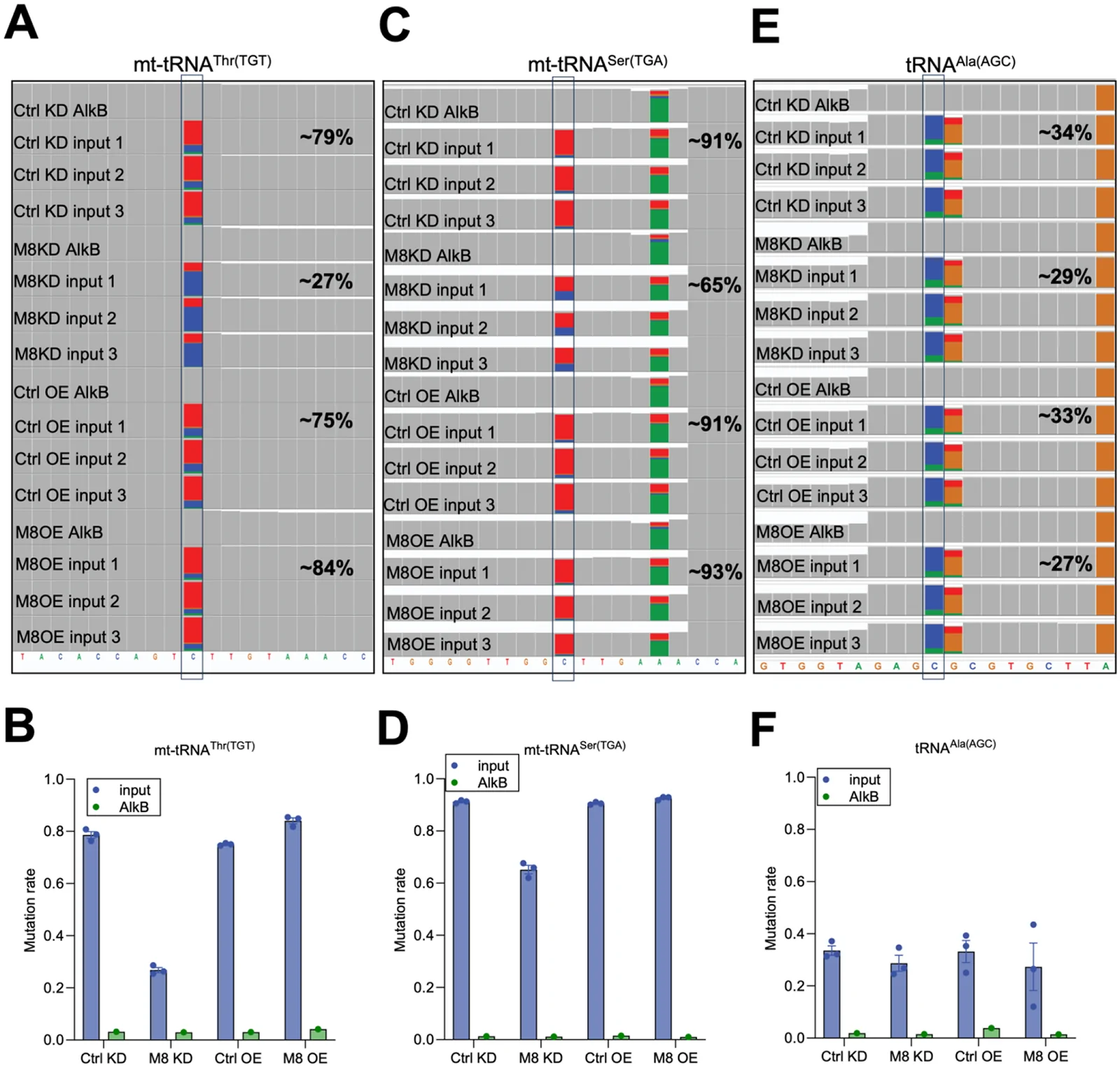
IGV visualization and mutation rates for selected m^3^C sites. IGV tracks (A) and mutation rates (B) for the mutation at the known m^3^C site in mt-tRNA^Thr(TGT)^ in input and AlkB-treated HeLa *METTL8* KD and OE samples with appropriate control samples. m^3^C site is highlighted in a rectangle and the average mutation in input libraries is listed. n=3 technical replicates. Mean and s.e.m. were plotted. IGV tracks (C) and mutation rates (D) for the mutation at the known m^3^C site in mt-tRNA^Ser(TGA)^ in input and AlkB-treated HeLa *METTL8* KD and OE samples with appropriate control samples. m^3^C site is highlighted in a rectangle and the average mutation in input libraries is listed. n=3 technical replicates. Mean and s.e.m. were plotted. IGV tracks (E) and mutation rates (F) for the mutation at the putative m^3^C site in mt-tRNA^Ala(AGC)^ in input and AlkB-treated HeLa METTL8 KD and OE samples with appropriate control samples. The putative m^3^C site is highlighted in a rectangle and the average mutation in input libraries is listed. n=3 technical replicates. Mean and s.e.m. were plotted.

**Table 1.** Comparison of m^3^C sites detected in this study and by selected other methods (HAC-seq, DM-tRNA-seq and HAMR). This table was adapted and modified from the literature.^15^

		Methods			
tRNA	Site	This study	HAC-seq	DM-tRNA-seq	HAMR
Ala^AGC^	C24	✓			
Arg^CCT^	C32	✓	✓	✓	
Arg^TCT^	C32	✓	✓	✓	✓
Leu^CAG^	C47d	✓	✓	✓	
Met^CAT^	C20	✓	✓	✓	✓
Ser^AGA^	C32	✓	✓	✓	
Ser^AGA^	C47d		✓	✓	
Ser^CGA^	C32	✓	✓	✓	✓
Ser^CGA^	C47d	✓	✓	✓	
Ser^GCT^	C32	✓	✓	✓	
Ser^GCT^	C47d	✓	✓	✓	
Ser^TGA^	C32	✓	✓	✓	✓
Ser^TGA^	C47d	✓	✓	✓	
Thr^AGT^	C32	✓	✓	✓	✓
Thr^CGT^	C32	✓	✓	✓	✓
Thr^TGT^	C32	✓	✓	✓	✓
mt-Met^CAT^	C32				✓
mt-Ser^TGA^	C32	✓	✓	✓	✓
mt-Thr^TGT^	C32	✓	✓	✓	✓

## Data Availability

Next-generation-sequencing (NGS) data for sh-Control and sh-*METTL8* poly(A)^+^ RNA and caRNA sequencing experiments are available at NCBI Gene Expression Omnibus, accession number GSE269470 (reviewer token: mfopuasiphwdret). Additional data is available at https://uchicago.box.com/s/3jkmgqom9iqef9ae6xbs70q9i553uuef (will be updated to GEO access prior to manuscript publication). Supplemental data files are listed below. Other data that support the findings presented in this study are available from the corresponding author upon request.
